# Supplementary material for: A comparative analysis of attitudes toward genome-edited food among Japanese public and scientific community
Source: PLoS One. 2024 Apr 16;19(4):e0300107. doi: 10.1371/journal.pone.0300107 (PMC11020778; doi:10.1371/journal.pone.0300107)
Supplement: S1 File — (DOCX) [file pone.0300107.s001.docx]

**Questionnaire on genome-edited foods**

February 2020

| This survey is conducted as part of a survey commissioned by the Ministry of Health, Labour and Welfare (MHLW) to investigate public awareness of genome-edited foods in Japan and to provide material for considering issues that will require further discussion.  We would appreciate it if you could answer as many of the questions as possible.  The results will be compiled in aggregate and your name and personal information will not be released to the public. The results will never be used for any purpose other than research and educational purposes.  If you have read the above information and agree to cooperate, please complete the questionnaire. Your response to this questionnaire will be deemed as your agreement to cooperate.  We sincerely appreciate your cooperation in this matter. |
| --- |

**Memo: This is a tentative version of the questionnaire translated into English.**

◆I would like to ask you about "genome-editing.”

Research on "genome-editing" is underway as a technology for pinpointing and precisely modifying genes. Foods created using this new genome-editing technology, which is different from genetically modified foods, are called "genome-edited foods. Currently, research is underway to create non-toxic potatoes and fast-growing sea bream.

Q1: Have you heard of keywords related to genome editing? (circle one)

1. （）I have heard of it and know it.
2. （）I have heard of it, but am not familiar with its content.
3. () I have never heard of it.

Q2: Do you think that How much do you think you know about genome-edited foods?

(circle one)

| I know it well. | be the one who knows | I know a little bit about it. | I can't say either way | I don't know much about it. | I know very little about it. | Never heard of it. |
| --- | --- | --- | --- | --- | --- | --- |
| 7 | 6 | 5 | 4 | 3 | 2 | 1 |

Q3: Do you agree with eating genome-edited food? (circle one).

| Strongly agree. | Agree. | Relatively agree. | I can't decide | Relatively disagree. | Disagree | strongly disagree |
| --- | --- | --- | --- | --- | --- | --- |
| 7 | 6 | 5 | 4 | 3 | 2 | 1 |

Q4: Can you trust experts’ discourses on safety of genome-edited foods? (circle one).

| Strongly agree. | Agree. | Relatively agree. | I can't decide | Relatively disagree. | Disagree | strongly disagree |
| --- | --- | --- | --- | --- | --- | --- |
| 7 | 6 | 5 | 4 | 3 | 2 | 1 |

Q5: Do you think genome-edited foods will be accepted in society? (circle one).

| Strongly agree. | Agree. | Relatively agree. | I can't decide | Relatively disagree. | Disagree | Strongly disagree |
| --- | --- | --- | --- | --- | --- | --- |
| 7 | 6 | 5 | 4 | 3 | 2 | 1 |

Q6: Which factors are important for the social acceptance of genome-edited food?

Please choose three factors from the list below that you think are highly important.

1. Interesting or not from scientific perspective
2. scientific validation
3. Whether society can prevent abuse and misuse by regulation
4. Whether genome-edited foods are necessary to the society
5. Seriousness of possible risks
6. Probability of possible risks
7. Whether experts can deal with risks
8. Credibility of research executors such as universities, countries, and companies, etc.
9. Clarification of responsibility and liability
10. Predictability of an genome-edited foods-powered future
11. There are no important things

( ) ・ ( ) ・ ( )

Please fill in the appropriate number of answers in the parentheses.

Q7:

What do you want to know?, Please choose three interesting topics.

1. Mechanisms
2. Benefits (advantages/good points)
3. Risks
4. Cost
5. Necessity
6. Industrial possibilities
7. Measures for safety
8. Ethical issues in genome editing
9. The way of Labeling
10. Schedule for future research activities on genome editing technology
11. Measures for cases of rumors about genome-edited foods
12. Measures for case for negative Impacts
13. National policy on regulations for genome-Edited Foods
14. Schedule for regulatory development
15. Current Status of international regulation
16. Nothing in particular that I need to know.
17. Other ( )

( ) ・ ( ) ・ ( )

Please fill in the appropriate response numbers in the parentheses.

Q8: Do you think humans can successfully use genome-edited foods without negative effects on the human body? (circle one)

| Strongly agree. | Agree. | Relatively agree. | I can't decide | Relatively disagree. | Disagree | Strongly disagree |
| --- | --- | --- | --- | --- | --- | --- |
| 7 | 6 | 5 | 4 | 3 | 2 | 1 |

Q9: Do you think humans can successfully use genome-edited foods in a way that does not have a negative impact on the environment? (circle one)

| Strongly agree. | Agree. | Relatively agree. | I can't decide | Relatively disagree. | Disagree | Strongly disagree |
| --- | --- | --- | --- | --- | --- | --- |
| 7 | 6 | 5 | 4 | 3 | 2 | 1 |

Q10: Do you think humans can successfully use genome-edited foods in a way that does not negatively impact the economy? (circle one)

| Strongly agree. | Agree. | Relatively agree. | I can't decide | Relatively disagree. | Disagree | Strongly disagree |
| --- | --- | --- | --- | --- | --- | --- |
| 7 | 6 | 5 | 4 | 3 | 2 | 1 |

Q11: Discussions on the labeling of genome-edited foods on food products have been progressing in Japan. Please select one statement from the following that best describes your thoughts about labeling genome-edited foods (circle one).

1. ( ) Genome-edited foods should be mandatorily labeled
2. ( ) Genome-edited foods should be labeled when nutritional composition are changed
3. ( ) Labeling of genome-edited foods should be left to the voluntary judgment of companies
4. ( ) Labeling genome-edited foods is not necessary
5. ( )Other ()
6. ( )I don't know.

Q12: When do you think genome-edited foods will be sold in supermarkets? Please choose one from the following items. (circle one)

1. ( ) Coming soon.
2. ( ) 1 year
3. ( ) Several years
4. ( ) At the earliest, aproximately 10 years.
5. () At the earliest, aproximately 20 years.
6. ( ) Even longer period
7. ( )I don't know.

Q13: Please circle the number that best reflects your opinion on the following issues related to genome-edited foods.

|  | I strongly agree. | Somewhat strongly agree. | Neither. | I don't think so. | I don't think so. |
| --- | --- | --- | --- | --- | --- |
| Useful for stable food supply | 5 | 4 | 3 | 2 | 1 |
| Helpful for people's health | 5 | 4 | 3 | 2 | 1 |
| Poses risks to people's health | 5 | 4 | 3 | 2 | 1 |
| Risks become apparent in the long run. | 5 | 4 | 3 | 2 | 1 |
| Good for Japan's economy. | 5 | 4 | 3 | 2 | 1 |
| Ecosystems of plants and insects are  changing | 5 | 4 | 3 | 2 | 1 |
| Insufficient confirmation of safety | 5 | 4 | 3 | 2 | 1 |
| There are unforeseen risks. | 5 | 4 | 3 | 2 | 1 |
| Technology can be misused. | 5 | 4 | 3 | 2 | 1 |
| I sense a bioethical problem. | 5 | 4 | 3 | 2 | 1 |
| Regulation is not likely to work. | 5 | 4 | 3 | 2 | 1 |
| I don't think we have a social  consensus on the use of genome-edited foods. | 5 | 4 | 3 | 2 | 1 |
| When something goes wrong with genome-  edited food, the government can't  handle it well. | 5 | 4 | 3 | 2 | 1 |
| When something goes wrong with genomeedited foods, companies don't deal with it well. | 5 | 4 | 3 | 2 | 1 |
| When something goes wrong with genomeedited foods, experts don't deal with it well. | 5 | 4 | 3 | 2 | 1 |
| I don't think the government will take responsibility if something goes wrong with genome edited foods. | 5 | 4 | 3 | 2 | 1 |
| I don't think the experts take  responsibility when something goes wrong with genome-edited foods. | 5 | 4 | 3 | 2 | 1 |
| I don't think companies will take  responsibility if something goes wrong with genome-edited foods. | 5 | 4 | 3 | 2 | 1 |
| I don't understand it well, and it's  kind of scary. | 5 | 4 | 3 | 2 | 1 |

Q14: Please select one statement from the following that best describes your thoughts about regulating genome-edited foods (circle one).

1. ( ) Genome-edited foods should be regulated by standards that reduce the risk as close to zero as possible.
2. ( ) Genome-edited foods should be regulated by scientifically reasonable standards.
3. ( ) Genome-edited foods should be regulated to the minimum necessary standards, with emphasis on economic efficiency
4. ( ) No need to regulate

Q15: We have asked you many questions about genome-edited foods. Please select one statement from the following that best describes your overall thoughts about genome-edited foods (circle one).

1. ( ) Although Safety concerns must be taken into account, we should press ahead with genome-edited foods.
2. ( ) I have a few concerns on safety, but it’s inevitable that genome-edited foods to be consumed.
3. ( ) It is better to focus on agricultural technology other than genome editing.
4. ( ) We shouldn’t press forward with genome-edited foods.
5. ( ) I don't know.

Q16: Please feel free to describe what you imagine regarding the risks of genome-edited foods. *Only keywords are acceptable.

( )

Q17: For each of the following items, please choose the degree to which you agree with the opinion. (circle one)

|  | I strongly agree. | Somewhat strongly agree. | Neither. | I don't think so. | I don't think so. |
| --- | --- | --- | --- | --- | --- |
| Eat as much local food as possible. | 5 | 4 | 3 | 2 | 1 |
| I want to eat a nutritionally  balanced diet. | 5 | 4 | 3 | 2 | 1 |
| I want agriculture to take  advantage of its local character. | 5 | 4 | 3 | 2 | 1 |
| I want to eat non-genetically  modified foods. | 5 | 4 | 3 | 2 | 1 |
| I want you to farm with care for  environmental conservation. | 5 | 4 | 3 | 2 | 1 |
| Seasonality and seasonality are important when choosing meals and foods. | 5 | 4 | 3 | 2 | 1 |
| When choosing food, I want to take care of the quality that can be  seen by appearance, such as  whether the food is damaged or not. | 5 | 4 | 3 | 2 | 1 |
| We want to strengthen the  relationship between agriculture and consumers. | 5 | 4 | 3 | 2 | 1 |
| I want to choose foods that show  production and cultivation  history. | 5 | 4 | 3 | 2 | 1 |
| It is favorable for agriculture to become more profitable through 6th industrialization and exports. | 5 | 4 | 3 | 2 | 1 |
| I would like to see more sales  where I can see what kind of farmer made the product. | 5 | 4 | 3 | 2 | 1 |
| I want them to be able to produce as many agricultural products as possible. | 5 | 4 | 3 | 2 | 1 |
| Eat as much natural, additivefree, organic, etc. food as  possible | 5 | 4 | 3 | 2 | 1 |
| I want farming to be done in a way  that is close to nature. | 5 | 4 | 3 | 2 | 1 |
| Price is important when choosing  meals and foods. | 5 | 4 | 3 | 2 | 1 |
| I'd like to see agriculture  incorporate new technologies. | 5 | 4 | 3 | 2 | 1 |
| Agricultural methods of production that consumers can afford at lower prices are desirable. | 5 | 4 | 3 | 2 | 1 |
| I want to eat well with processed  foods, prepared foods, boxed  lunches, eating out, etc. | 5 | 4 | 3 | 2 | 1 |
| Want to select famous branded food  products (e.g., Yubari melon,  Matsuzaka beef, etc.) | 5 | 4 | 3 | 2 | 1 |

Q18: Please indicate whether the following statement is correct or incorrect by placing a circle or an X in parentheses.

1. The oxygen we use to breathe comes from plants ( )
2. The gene that determines whether a baby will be a girl or not is the gene that the mother has ( )
3. Antibiotics kill viruses as well as bacteria.
4. Present-day humans evolved from a primitive animal species ( )
5. Radioactive milk is safe if boiled ( )
6. Some bacteria can live in wastewater ( )
7. It is possible to find out in the second or third trimester of pregnancy whether a baby will have Down syndrome ( )
8. The yeast that brews beer is made up of living microorganisms ( )
9. More than half of human genes are identical to those of chimpanzees ( )
10. By eating genetically modified fruits, a person's genes are also modified ( )
11. To clone (duplicate) an organism is to produce genetically identical offspring ( )
12. Ordinary tomatoes do not contain the gene, but genetically modified tomatoes do ( )
13. Genetically modified animals are always larger than normal animals ( )
14. Animal genes cannot be introduced into plants ( )

Q19: To what extent do you think the following attitudes and ideas apply to you? (One of each)

|  | I strongly agree. | Somewhat strongly agree. | Neither. | I don't think so. | I don't think so. |
| --- | --- | --- | --- | --- | --- |
| I'm more of a newspaper reader. | 5 | 4 | 3 | 2 | 1 |
| I watch/listen to TV/radio a lot. | 5 | 4 | 3 | 2 | 1 |
| I'm more of a book reader. | 5 | 4 | 3 | 2 | 1 |
| I use the Internet a lot. | 5 | 4 | 3 | 2 | 1 |
| I often exchange information with family and friends. | 5 | 4 | 3 | 2 | 1 |
| I usually do a lot of research on things I don't understand or need to know in my life. | 5 | 4 | 3 | 2 | 1 |
| When I want to know about a hot topic in society, I often look it up. | 5 | 4 | 3 | 2 | 1 |
| When someone close to you becomes ill, research the treatment and cause of the illness on your own. | 5 | 4 | 3 | 2 | 1 |
| I'm a frequent visitor to museums, science museums and public lectures. | 5 | 4 | 3 | 2 | 1 |
| Easy to believe people's stories | 5 | 4 | 3 | 2 | 1 |
| I don't get carried away by the atmosphere around me. | 5 | 4 | 3 | 2 | 1 |
| Able to talk with others about problems that need to be solved and solve them well | 5 | 4 | 3 | 2 | 1 |
| When solving a problem, it's more important to "make sense" than to "make it go away. | 5 | 4 | 3 | 2 | 1 |
| Interested in discussing social and other  public issues | 5 | 4 | 3 | 2 | 1 |
| I'm an active participant in community and civic activities. | 5 | 4 | 3 | 2 | 1 |
| I go to elections as much as I can. | 5 | 4 | 3 | 2 | 1 |
| I want to participate as a citizen in scientific and technological evaluation activities | 5 | 4 | 3 | 2 | 1 |
| Supernatural phenomena like ESP do exist. | 5 | 4 | 3 | 2 | 1 |
| They're good at fixing and repairing broken things. | 5 | 4 | 3 | 2 | 1 |
| As soon as a new technological appliance goes on sale, they want it. | 5 | 4 | 3 | 2 | 1 |
| I like making things (including cooking, gardening, crafts, etc.) | 5 | 4 | 3 | 2 | 1 |
| Be able to use new electronic equipment quickly | 5 | 4 | 3 | 2 | 1 |
| I have a wealth of knowledge about science and technology. | 5 | 4 | 3 | 2 | 1 |
| Learn more about science and technology | 5 | 4 | 3 | 2 | 1 |
| I want to contribute to the global environment. | 5 | 4 | 3 | 2 | 1 |
| I'm more likely to put plastic bottles and bottles in the recycling. | 5 | 4 | 3 | 2 | 1 |
| Even if the price is a little higher, it is possible to use non-wasteful products and products that consume less electricity and fuel. I'm the one who buys less. | 5 | 4 | 3 | 2 | 1 |
| I'm good at sports. | 5 | 4 | 3 | 2 | 1 |
| Good sense of sound and rhythm | 5 | 4 | 3 | 2 | 1 |
| I'm good at predicting a three-dimensional image from a development (plane). | 5 | 4 | 3 | 2 | 1 |
| I'm good at reading maps. | 5 | 4 | 3 | 2 | 1 |
| I'm good at getting to the point of long sentences, lectures, etc. | 5 | 4 | 3 | 2 | 1 |
| I'm good at thinking things through logically. | 5 | 4 | 3 | 2 | 1 |
| I'm good at capturing what things have in common. | 5 | 4 | 3 | 2 | 1 |
| I often think back and reflect on my decisions and judgments | 5 | 4 | 3 | 2 | 1 |

Q20: What do you think of the following opinions/thoughts about science and society. please choose one from each of the four options. (One of each)

|  | I strongly agree. | Somewhat strongly agree. | Neither. | I don't think so. | I don't think so. |
| --- | --- | --- | --- | --- | --- |
| 1 Understanding of science and technology is useful in daily life | 5 | 4 | 3 | 2 | 1 |
| 2 Science has value not only for its usefulness but also for its intellectual enjoyment. | 5 | 4 | 3 | 2 | 1 |
| 3 Scientific discoveries and development of new technologies enrich society and people. | 5 | 4 | 3 | 2 | 1 |
| 4 The development of science and technology is necessary for a country to achieve international development. | 5 | 4 | 3 | 2 | 1 |
| 5 Science and technology have negative  effects on society and human beings | 5 | 4 | 3 | 2 | 1 |
| 6 The viewpoints of society and citizens need to be reflected in the state of science and technology | 5 | 4 | 3 | 2 | 1 |
| 7 It would be good if scientific thinking  permeates society. | 5 | 4 | 3 | 2 | 1 |
| 8 With the development of technology, it is possible to make products and things that do not have negative social impacts. | 5 | 4 | 3 | 2 | 1 |
| 9 We should take a hard look at false ideas and products masquerading as science. | 5 | 4 | 3 | 2 | 1 |
| 10 Scientists and engineers are doing research with the idea of improving our lives. | 5 | 4 | 3 | 2 | 1 |
| 11 Scientists and engineers are reliable | 5 | 4 | 3 | 2 | 1 |
| 12 Science and technology can be left to  scientists and engineers | 5 | 4 | 3 | 2 | 1 |
| 13 Politicians and government agencies are trustworthy | 5 | 4 | 3 | 2 | 1 |
| 14 Politics and government can be left to politicians and government agencies | 5 | 4 | 3 | 2 | 1 |

Finally, let me ask you a few questions about yourself

F1 Please tell us about your child(ren). (circle one)

- 1. childless
  2. With children (including pregnant) → ( ) (Please indicate the number of children)

[Asked of those who chose "2 with children" in F1].

F1-2 What is the age of your youngest child?

age counter for years

F2 Please indicate your household's total income (annual and face value) for the last year. (circle one)

| 1. Less than 3 million yen 2. Less than 3-4 million yen 3. Less than 4-6 million yen 4. Less than 6-8 million yen 5. Less than 8-10 million yen | 1. Less than 10-12 million yen 2. Less than 12-15 million yen 3. Less than 15-20 million yen 4. Less than 20-30 million yen 10 More than 30 million yen |
| --- | --- |

F3 Which of the following schools did you last graduate? (circle one)

( ) Junior high school

( ) Secondary school (operated under postwar guidelines)

( ) High school

( ) High school (operated under postwar guidelines)

( ) Vocational school

( ) College of technology and junior college

( ) University

( ) Graduate School

( ) Onter (Specify: )

F4 Please indicate the area(s) in which you feel you have learned the most.

( ) Science

( ) Humanities and Social Science

( ) Other ( )

F5. Regarding your current occupation, which of the following areas are you classified as your area of expertise?

( ) Science

( ) Humanities

( ) Agriculture and Food

( ) Other ( )

( ) I don't know

This is the end of the questionnaire. Thank you very much for your cooperation.

◇◇

**Memo: Below is for experts**

Questionnaire on genome-edited foods

March 2020

| This survey is part of a study commissioned by the Ministry of Health, Labour and Welfare (MHLW), 'Research to ensure the safety and risk communication of foods obtained using new biotechnologies', to investigate attitudes towards genome-edited foods in Japan. The survey aims to identify issues for future risk communication by comparing the responses of the general public with those of experts and research professionals.  We would appreciate it if you could answer as many of the questions as possible.  The results will be compiled in aggregate and your name and personal information will not be released to the public. The results will never be used for any purpose other than research and educational purposes.  If you have read the above information and agree to cooperate, please complete the questionnaire. Your response to this questionnaire will be deemed as your agreement to cooperate.  We sincerely appreciate your cooperation in this matter. |
| --- |

Q0. Please indicate your gender (optional answers).

( ) male

( ) female

Q0. Please indicate your age (optional answer).

( )

◆I would like to ask you about "genome editing.”

Research on "genome editing" is underway as a technology for pinpointing and precisely modifying genes. Foods created using this new genome-editing technology, which is different from genetically modified foods, are called "genome-edited foods. Currently, research is underway to create non-toxic potatoes and fast-growing sea bream.

Q1: Have you heard of keywords related to genome editing? (circle one)

( ) I have heard of it and know it.

( ) I have heard of it, but am not familiar with its content.

( ) I have never heard of it.

Q2: Do you think that how much do you think you know about genome-edited foods?

(circle one)

| I know it well. | be the one who knows | I know a little bit about it. | I can't say either way | I don't know much about it. | I know very little about it. | Never heard of it. |
| --- | --- | --- | --- | --- | --- | --- |
| 7 | 6 | 5 | 4 | 3 | 2 | 1 |

Q3: Do you agree with eating genome-edited food? (circle one).

| Strongly agree. | Agree. | Relatively agree. | I can't decide | Relatively disagree. | Disagree | strongly disagree |
| --- | --- | --- | --- | --- | --- | --- |
| 7 | 6 | 5 | 4 | 3 | 2 | 1 |

Q4: Can you trust experts’ discourses on safety of genome-edited foods? (circle one).

| Strongly agree. | Agree. | Relatively agree. | I can't decide | Relatively disagree. | Disagree | strongly disagree |
| --- | --- | --- | --- | --- | --- | --- |
| 7 | 6 | 5 | 4 | 3 | 2 | 1 |

Q5: Do you think genome-edited foods will be accepted in society? (circle one).

| Strongly agree. | Agree. | Relatively agree. | I can't decide | Relatively disagree. | Disagree | strongly disagree |
| --- | --- | --- | --- | --- | --- | --- |
| 7 | 6 | 5 | 4 | 3 | 2 | 1 |

Q6: Which factors are important for the social acceptance of genome-edited food?

Please choose three factors from the list below that you think are highly important.

1. Interesting or not from scientific perspective
2. scientific validation
3. Whether society can prevent abuse and misuse by regulation
4. Whether genome-edited foods are necessary to the society
5. Seriousness of possible risks
6. Probability of possible risks
7. Whether experts can deal with risks
8. Credibility of research executors such as universities, countries, and companies, etc.
9. Clarification of responsibility and liability
10. Predictability of an genome-edited foods-powered future
11. There are no important things

( ) ・ ( ) ・ ( )

Please fill in the appropriate number of answers in the parentheses.

Q7: What do you want to inform? Please choose three interesting topics.

1. Mechanisms
2. Benefits (advantages/good points)
3. Risks
4. Cost
5. Necessity
6. Industrial possiblities
7. Measures for safety
8. Ethical issues in genome editing
9. The way of Labeling
10. Schedule for future research activities on genome editing technology
11. Measures for cases of rumors about genome-edited foods
12. Measures for case for negative Impacts
13. National policy on regulations for genome-Edited Foods
14. Schedule for regulatory development
15. Current Status of international regulation
16. Nothing in particular that I need to know.
17. Other ( )

( ) ・ ( ) ・ ( )

Please fill in the appropriate response numbers in the parentheses.

Q8:-1 Do you think humans can successfully use genome-edited foods without negative effects on the human body? (circle one)

| I strongly agree. | I think so. | or rather  I think so | I can't say either way | I rather disagree. | I don't think so. | I don't think so at all. |
| --- | --- | --- | --- | --- | --- | --- |
| 7 | 6 | 5 | 4 | 3 | 2 | 1 |

Q8:-2: Do you think humans can successfully use genome-edited foods in a way that does not have a negative impact on the environment? (circle one)

| I strongly agree. | I think so. | or rather  I think so | I can't say either way | I rather disagree. | I don't think so. | I don't think so at all. |
| --- | --- | --- | --- | --- | --- | --- |
| 7 | 6 | 5 | 4 | 3 | 2 | 1 |

Q8:-3: Do you think humans can successfully use genome-edited foods in a way that does not negatively impact the economy? (circle one)

| I strongly agree. | I think so. | or rather  I think so | I can't say either way | (I) rather disagree | I don't think so. | I don't think so at all. |
| --- | --- | --- | --- | --- | --- | --- |
| 7 | 6 | 5 | 4 | 3 | 2 | 1 |

Q9: Discussions on the labeling of genome-edited foods on food products have been progressing in Japan. Please select one statement from the following that best describes your thoughts about labeling genome-edited foods (circle one).

( ) Genome-edited foods should be mandatorily labeled

( ) Genome-edited foods should be labeled when nutritional composition are changed

( ) Labeling of genome-edited foods should be left to the voluntary judgment of companies

( ) Labeling genome-edited foods is not necessary

( )Other ( )

( )I don't know.

Q10: When do you think genome-edited foods will actually be sold in supermarkets? Please choose one from the following items. (circle one)

( ) Coming soon.

( ) 1 year

( ) Several Years

( ) At the earliest, approximately 10 years.

( ) At the earliest, approximately 20 years.

( ) Even longer period

( ) I don't know.

Q11: Please circle the number that best reflects your opinion on the following issues related to genome-edited foods.

|  | I strongly agree. | Somewhat strongly agree. | Neither. | I don't think so. | I don't think so. |
| --- | --- | --- | --- | --- | --- |
| Useful for stable food supply | 5 | 4 | 3 | 2 | 1 |
| Helpful for people's health | 5 | 4 | 3 | 2 | 1 |
| Poses risks to people's health | 5 | 4 | 3 | 2 | 1 |
| Risks become apparent in the long run. | 5 | 4 | 3 | 2 | 1 |
| Good for Japan's economy. | 5 | 4 | 3 | 2 | 1 |
| Ecosystems of plants and insects are  changing | 5 | 4 | 3 | 2 | 1 |
| Insufficient confirmation of safety | 5 | 4 | 3 | 2 | 1 |
| There are unforeseen risks. | 5 | 4 | 3 | 2 | 1 |
| Technology can be misused. | 5 | 4 | 3 | 2 | 1 |
| I sense a bioethical problem. | 5 | 4 | 3 | 2 | 1 |
| Regulation is not likely to work. | 5 | 4 | 3 | 2 | 1 |
| I don't think we have a social  consensus on the use of genome-edited foods. | 5 | 4 | 3 | 2 | 1 |
| When something goes wrong with genome-  edited food, the government can't  handle it well. | 5 | 4 | 3 | 2 | 1 |
| When something goes wrong with genomeedited foods, companies don't deal with it well. | 5 | 4 | 3 | 2 | 1 |
| When something goes wrong with genomeedited foods, experts don't deal with it well. | 5 | 4 | 3 | 2 | 1 |
| I don't think the government will take responsibility if something goes wrong with genome edited foods. | 5 | 4 | 3 | 2 | 1 |
| I don't think the experts take  responsibility when something goes wrong with genome-edited foods. | 5 | 4 | 3 | 2 | 1 |
| I don't think companies will take  responsibility if something goes wrong with genome-edited foods. | 5 | 4 | 3 | 2 | 1 |
| I don't understand it well, and it's  kind of scary. | 5 | 4 | 3 | 2 | 1 |

Q12: Please select one statement from the following that best describes your thoughts about regulating genome-edited foods (circle one).

( ) Genome-edited foods should be regulated by standards that reduce the risk as close to zero as possible.

( ) Genome-edited foods should be regulated by scientifically reasonable standards.

( ) Genome-edited foods should be regulated to the minimum necessary standards, with emphasis on economic efficiency

( ) No need to regulate

Q13: We have asked you many questions about genome-edited foods. Please select one statement from the following that best describes your overall thoughts about genome-edited foods (circle one).

( ) Although safety concerns must be taken into account, we should press ahead with genome-edited foods.

( ) I have a few concerns on safety, but it’s inevitable that genome-edited foods to be consumed.

( ) It is better to focus on agricultural technology other than genome editing.

( ) We shouldn’t press forward with genome-edited foods.

( ) I don't know.

Q14: Please feel free to describe what you imagine regarding the risks of genome-edited foods. *Only keywords are acceptable.

( )

Q15: For each of the following items, please choose the degree to which you agree with the opinion. (circle one)

|  | I strongly agree. | Somewhat strongly agree. | Neither. | I don't think so. | I don't think so. |
| --- | --- | --- | --- | --- | --- |
| Eat as much local food as possible. | 5 | 4 | 3 | 2 | 1 |
| I want to eat a nutritionally  balanced diet. | 5 | 4 | 3 | 2 | 1 |
| I want agriculture to take  advantage of its local character. | 5 | 4 | 3 | 2 | 1 |
| I want to eat non-genetically  modified foods. | 5 | 4 | 3 | 2 | 1 |
| I want you to farm with care for  environmental conservation. | 5 | 4 | 3 | 2 | 1 |
| Seasonality and seasonality are important when choosing meals and foods. | 5 | 4 | 3 | 2 | 1 |
| When choosing food, I want to take care of the quality that can be  seen by appearance, such as  whether the food is damaged or not. | 5 | 4 | 3 | 2 | 1 |
| We want to strengthen the  relationship between agriculture and consumers. | 5 | 4 | 3 | 2 | 1 |
| I want to choose foods that show  production and cultivation  history. | 5 | 4 | 3 | 2 | 1 |
| It is favorable for agriculture to become more profitable through 6th industrialization and exports. | 5 | 4 | 3 | 2 | 1 |
| I would like to see more sales  where I can see what kind of farmer made the product. | 5 | 4 | 3 | 2 | 1 |
| I want them to be able to produce as many agricultural products as possible. | 5 | 4 | 3 | 2 | 1 |
| Eat as much natural, additivefree, organic, etc. food as  possible | 5 | 4 | 3 | 2 | 1 |
| I want farming to be done in a way  that is close to nature. | 5 | 4 | 3 | 2 | 1 |
| Price is important when choosing  meals and foods. | 5 | 4 | 3 | 2 | 1 |
| I'd like to see agriculture  incorporate new technologies. | 5 | 4 | 3 | 2 | 1 |
| Agricultural methods of production that consumers can afford at lower prices are desirable. | 5 | 4 | 3 | 2 | 1 |
| I want to eat well with processed  foods, prepared foods, boxed  lunches, eating out, etc. | 5 | 4 | 3 | 2 | 1 |
| Want to select famous branded food  products (e.g., Yubari melon,  Matsuzaka beef, etc.) | 5 | 4 | 3 | 2 | 1 |

Finally, let me ask you a few questions about yourself

Q16. Which of the following schools did you last graduate? (circle one)

( ) Junior high school

( ) Secondary school (operated under postwar guidelines)

( ) High school

( ) High school (operated under postwar guidelines)

( ) Vocational school

( ) College of technology and junior college

( ) University

( ) Graduate School

( ) Onter (Specify: )

Q17. Please indicate the area(s) you feel you have learned the most?

( ) Science

( ) Humanities and Social Science

( ) Other ( )

Q18. Regarding your current occupation, which of the following areas are you classified as your area of expertise?

( ) Science

( ) Humanities

( ) Agriculture and Food

( ) Other ( )

( ) I don't know

Q19. Please indicate your area of expertise (e.g., field or subdivision of Grantin-Aid for Scientific Research, keywords, etc.).

( )

This is the end of the questionnaire. Thank you very much for your cooperation.

◇◇
