# Supplementary material for: A comparative analysis of attitudes toward genome-edited food among Japanese public and scientific community
Source: PLoS One. 2024 Apr 16;19(4):e0300107. doi: 10.1371/journal.pone.0300107 (PMC11020778; doi:10.1371/journal.pone.0300107)
Supplement: S1 Table — (DOCX) [file pone.0300107.s003.docx]

**Table Sup1: Peason’s Correlation between the** **attitudes toward GEF.**

|  | 1-b | 1-c | 1-d |
| --- | --- | --- | --- |
| 1-b Opinions on the social acceptance of GEF | 1 | .512** | .576** |
| 1-c Degree of agreement with eating GEF | .512** | 1 | .736** |
| 1-d Trust-worthiness of experts on GEF | .576** | .736** | 1 |
|  |  |  |  |
