# Supplementary material for: A comparative analysis of attitudes toward genome-edited food among Japanese public and scientific community
Source: PLoS One. 2024 Apr 16;19(4):e0300107. doi: 10.1371/journal.pone.0300107 (PMC11020778; doi:10.1371/journal.pone.0300107)
Supplement: Attachment — Submitted filename: Response to reviewer.docx [file pone.0300107.s004.docx]

**Response to reviewer’s comment**

We would like to appreciate to editors and reviewers for their kind cooperation and valuable opinions to our manuscript. We would like to show summary of our revision for reviewer’s comment here. In following text, reviewer comments were shown with **Bold**, and added or revised sentence in new revised manuscript were highlighted in red characters.

**Reviewer 1:**

1. **In general, the intricacy of the survey is not clearly transmitted. Perhaps the data presentation is ambiguous but the depth of questions asked needs to be improved. For instance, is the question from Fig 1a “Recognition of keywords related to GEF (have you heard of keywords related to GEF?)” the only question asked to determine the public’s recognition of GEF or were there follow-up questions to assess the respondents’ responses? In addition, it is not clear if the respondents were given a list of keywords which they were asked to recognize, or were the respondents asked if they had heard of any keywords related to GEF? Similarly, in Fig 2, were the respondents given an example of a label or information that should appear on a label? A full version of the (translated) survey should be made available, i.e., in supplemental data for readers to better grasp the questions asked, hence the extent of the study conducted.**

Answer

In order to make clear the contents and structure of questionnaire, we added English translation of key questionnaire (tentative version) as supplemental file.

2. **It would be informative to also have a breakdown of the percentage of respondents leading from one question to another to improve the depth of analysis. For instance, what percentage of respondents who “cannot decide” the social acceptance of GEF (Fig 1b) would or would not consume GEF (Fig 1c), and of which, how many were related to trust issues (Fig 1c)? Additionally, how does the degree of trustworthiness (Fig 1c) relate to the information the respondents are interested in (Fig 3)? What is the percentage of well-informed public are acceptive of GEF? Drawing some careful correlation analysis might provide more information and improve discussion.**

Answer

We appreciate to this suggestion. We added some analysis and description of results.

Firstly we added results of correlation of questions shown in Fig1(b) to Fig(1d), and added sentence after the Fig1. This result is shown as Sup2.

There are strong correlations between the answer pattern of the three questions on attitudes toward GEF (See Sup. 2). Generally, the stronger respondents think GEF will be accepted in society, the stronger they agree with eating GEF. Simultaneously, they also show their trust in expert’s discourses on the safety of GEF.

In addition, we also conducted cross-tabulation between trust-worthiness of expert’s on GEF (see Fig. 1(d)), Important factors for acceptance, and interested topics. These results were shown as Sup3 and 4. Description of result were written as below and added after the Fig 4.

We also conducted cross tabulation of public responses between questions on the trust-worthiness of experts on GEF (see Fig. 1(d)), important factors for acceptance, and interested topics (see Sup. 3 and 4). Generally, we found that people who trust expert’s discourses on safety regard scientific validation, necessity, and the prevention of abuse and misuse by regulation as key factors for social acceptance of GEF. They also would like to know the mechanism, benefit, and cost compared to another group. However, people who do not trust expert’s discourses on safety regard risk and clarification of responsibility and liability as key factors for social acceptance of GEF. They would like to know measures for negative effects, national policy or regulation, and ethical issues.

3. **Since the Japanese Society of Genome Editing is open to both academic and industry experts, authors should indicate if the experts are from the public or private sector. Reanalysis of the data taking the private or public sector into consideration would provide another perspective. The reason for this is to take into consideration the possibility of bias, i.e., that the private sector could be profit-driven.**

Answer

The survey did not ask about the occupational sector, so the exact numbers are not known. However, the majority of genome editing society and the used mailing list were academia.

4. **In agreement with the authors’ awareness of the limitations of this survey, perhaps difficult but a follow-up on the public’s acceptance of GEF after the release of GEF products into the Japanese market would be highly interesting. While this request might be out of scope, discussion towards this direction would be appreciated. A helpful literature:** [**https://doi.org/10.3390/su15129662**](https://doi.org/10.3390/su15129662)

Answer

We appreciate great suggestion. We added the literature below.

Shigi, R.; Seo, Y. Consumer Acceptance of Genome-Edited Foods in Japan. Sustainability 2023, 15, 9662. https://doi.org/10.3390/su15129662

In addition, we revise our introduction and discussion, considering implication of those literatures. We added sentences below:

A recent literature, after the onset GEF commercialization, showed consumer acceptance of GEF—with 550 respondents [44]. It was found that an interaction existed between awareness of genome-editing technology, information credibility, perceived usefulness, and willingness to buy GEF [44].

And then, we added sentence below to discussion:

However, there is a pertinent need to provide the public with basic information on GEF, considering the potential effects of information sharing on the mechanisms and benefits of GEF suggested by previous studies [44].

**Minor comments:
Minor-1: Line 1: Nobel prize**

Answer: We revised typos.

**Minor-2: Introduction could be improved, e.g. line 67-71: More elaboration on what are the 3 categories of GEF, and what are the 2 categories of regulation- how are they differently regulated?**

Answer

We added explanation about three categories of GEF and their regulatory treatment. We added paragraphs as below in Introduction.

Within the Japanese regulatory system on research and development, genome editing is differently controlled by three types of editing: SDN-1, SDN-2, and SDN-3. SDN-1 is the technique of introducing a break at a target site in the genome. SDN-2 is the addition of a few base pairs of nucleotides to the target site. SDN-3 is introducing a gene at the target site. These types of gene editing are differently regulated according to the viewpoints of environmental assessment and food sanitation [9, 12].

For field cultivation of genome-edited crops, they are regulated mainly by the Ministry of Environment (MoE) and Ministry of Agriculture, Forestry, and Fishery (MAFF), and their discussions are related to issues of environmental assessment and biodiversity. In this field cultivation, categories of SND-2 and SDN-3 of genome-edited crops (or animals) are regulated under the Cartagena Domestic Law enacted in conformance with the Cartagena Protocol [9, 12].

**Minor-3: Table 1: Under Education “High/Graduate School” should be “High/Graduate school”. Under Annual Income, “4 millionn” should be “4 million”**

Answer: We revised typos.

**Minor-4: In general, language support would facilitate a smoother reading process.**

Answer: Our manuscript were native checked by Editage. We attached certification.

**Reviewer 2:**

1. **In abstract, a number of general public is given 3000, whereas this number is 4000 in the methods. Please correct it.**
2. **Correct Important word in Table 2.**

Answer: We revised typos.
