# Supplementary material for: A comparative analysis of attitudes toward genome-edited food among Japanese public and scientific community
Source: PLoS One. 2024 Apr 16;19(4):e0300107. doi: 10.1371/journal.pone.0300107 (PMC11020778; doi:10.1371/journal.pone.0300107)
Supplement: Attachment — Submitted filename: PONE-D-23-28736_R1.docx [file pone.0300107.s005.docx]

PONE-D-23-28736-R2

The authors have addressed most of the comments made significant modifications to the manuscript. I especially appreciate Supplemental Table S2 and Table S3 and I invite the authors to consider making Tables S2 and S3 as main tables and/or discuss them more heavily in the Results/Discussion. There are a few minor details which require polishing before I can recommend for acceptance.

Minor

Line 107: SDN-2, not SND-2

Line 146: Please change “images” to “impression”

Line 324: Please check if ‘sup. 3’ is supposed to be Table S2. Provide table legends for Table S2 and Table S3. Please change “Generally, we found that people who trust..” to “Generally, we found that people trust..”

Line 327: Please revise this line to make the message clearer.

Line 328: Please change “people who do not trust..” to “people do not trust”

Line 330-332: Please revise this line to make the message clearer.

Line 322-331: Please use quotation marks for each factor, i.e. “scientific validation”, “necessity” etc, to ease reading. Consider revising this paragraph- message not easily understood.

All figures : Make sure the legends in the main text are similar to those used in the figures.

Methods: Please describe how statistics and correlation analysis were performed.
