# Supplementary material for: A comparative analysis of attitudes toward genome-edited food among Japanese public and scientific community
Source: PLoS One. 2024 Apr 16;19(4):e0300107. doi: 10.1371/journal.pone.0300107 (PMC11020778; doi:10.1371/journal.pone.0300107)
Supplement: Attachment — Submitted filename: Response to reviewer_20240215.docx [file pone.0300107.s006.docx]

**Response to reviewer’s comment**

We would like to appreciate to reviewers for their valuable opinions to our manuscript. We would like to show summary of our revision for reviewer’s comment here. In following text, reviewer comments were shown with **Bold**.

**Reviewer 1:**

**The authors have addressed most of the comments made significant modifications to the manuscript. I especially appreciate Supplemental Table S2 and Table S3 and I invite the authors to consider making Tables S2 and S3 as main tables and/or discuss them more heavily in the Results/Discussion. There are a few minor details which require polishing before I can recommend for acceptance.**

Answer: We revised our manuscript according to this reviewer’s suggestion. We moved Table S2 and S3 to main Tables (as Table 3 & 4), and added below sentences in the “Discussion” section.

Simultaneously, we need to consider the different attitudes between the two types of groups, with or without trust in experts’ discourses on safety. Tables 3 and 4 show that the two groups have different interests and concerns regarding GEF. People who trust expert’s discourses on safety generally have interests in scientific contents and “the way of prevention of abuse and misuse by regulation.” On the other hand, another group have interests in issues related risk governance such as “whether experts can deal with risks,” “clarification of responsibility and liability,” “national policy or regulation,” “ethical issues,” and so on. We should not see the public as a monolith. We need to consider what kinds of information and factors are of interest and expected in each group.

**Minor**

**Line 107: SDN-2, not SND-2**

Answer: We revised typos.

**Line 146: Please change “images” to “impression”**

Answer: We revised it.

**Line 324: Please check if ‘sup. 3’ is supposed to be Table S2. Provide table legends for Table S2 and Table S3. Please change “Generally, we found that people who trust..” to “Generally, we found that people trust..”**

Answer: We revised it. And we added table legends to Table S2 and Table S3. In addition, we move supplemental tables to main tables according to reviewer’s suggestion. Revised manuscripts are also shown above.

**Line 327: Please revise this line to make the message clearer.**

Answer: We revised this line as below:

For more effective communication between the public and experts on GEF,

**Line 328: Please change “people who do not trust..” to “people do not trust”**

Answer: We think relative pronoun “who” is necessary here. We revised paragraphs around this line according to previous reviewer’s suggestion regarding Table S2 and S3. Revised sentences are shown in before and file with markup.

**Line 330-332: Please revise this line to make the message clearer.**

Answer: We revised this line as below:

It is important to provide basic information on GEF to the public continuously, considering the potential effects of information-sharing on the mechanisms and benefits of GEF, as suggested by previous studies.

**Line 322-331: Please use quotation marks for each factor, i.e. “scientific validation”, “necessity” etc, to ease reading. Consider revising this paragraph- message not easily understood.**

Answer: We revised it.

**All figures : Make sure the legends in the main text are similar to those used in the figures.**

Answer: We re-checked and revised them.

**Methods: Please describe how statistics and correlation analysis were performed.**

Answer: We added sentence below in the “Methods” section.

In our statistical analysis, we conducted a chi-square test to determine independence. In addition, we calculated Pearson’s correlation (see Table 3). For our analysis, we used SPSS ver. 26.
